# Synthesis and glycosidase inhibitory activity of new hexa-substituted C8-glycomimetics

**DOI:** 10.1186/1860-5397-1-12

**Published:** 2005-10-07

**Authors:** Olivia Andriuzzi, Christine Gravier-Pelletier, Gildas Bertho, Thierry Prangé, Yves Le Merrer

**Affiliations:** 1 Université Paris Descartes, UMR 8601 CNRS, Laboratoire de Chimie et Biochimie Pharmacologiques et Toxicologiques, 45 rue des Saints-Pères, 75270 Paris Cedex 06, France; 2 Université Paris Descartes, UMR 8015 CNRS, Laboratoire de Cristallographie et RMN Biologiques, 4 avenue de l'Observatoire, 75270 Paris Cedex 06, France

## Abstract

**Background:**

Glycosidases are involved in several metabolic pathways and the development of inhibitors is an important challenge towards the treatment of diseases, such as diabetes, cancer and viral infections including AIDS. Thus, inhibition of intestinal α-glucosidases can be used to treat diabetes through the lowering of blood glucose levels, and α-glucosidase inhibitors are being marketed against type 2 (non-insulinodependent *mellitus*) diabetes (*i.e*.: Glyset^®^ or Diastabol^®^, Basen^®^ and Glucor^®^ or Precose^®^).

**Results:**

In that context, new C8-carbasugars and related aminocyclitols have been targeted in order to study the effect of the enhanced flexibility and of the new spatial distribution displayed by these structures on their adaptability in the active site of the enzymes. The synthesis of these new C8-glycomimetics is described from enantiomerically pure C_2_-symmetrical polyhydroxylated cyclooctenes. Their obtention notably involved a *syn*-dihydroxylation, and more extended functionalization through formation of a *cis*-cyclic sulfate followed by amination and subsequent reductive amination. This strategy involving the nucleophilic opening of a *cis*-cyclic sulfate by sodium azide is to our knowledge the first example in C8-series. It revealead to be an efficient alternative to the nucleoplilic opening of an epoxide moiety which proved unsuccessful in this particular case, due to the hindered conformation of such epoxides as demonstrated by X-ray cristallographic analysis.

**Conclusion:**

The biological activity of the synthesized glycomimetics has been evaluated towards 24 commercially available glycosidases. The weak observed activities can probably be related to the spatial disposition of the hydroxy and amino groups which depart too much from that realized in glycomimetics such as valiolamine, voglibose and valienamine. Nevertheless, the synthetic strategy described here is efficient and general, and could be extended to increase the diversity of the glycosidase inhibitors obtained since this diversity is introduced in an ultimate step of the synthesis.

## Introduction

**Figure 1:**
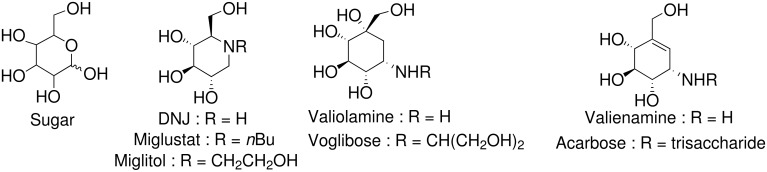
Sugars, iminosugars and carbasugars.

There is a considerable interest in the design of molecules able to mimic carbohydrates which play critical roles in various biological events such as for example, cell-cell recognition and adhesion, cell growth and differentiation.[1–9] In this context, the goal is to obtain new compounds with improved efficacy, stability and specificity. Thus, a change from an aldopyranoside to a 1-deoxy-iminosugar (Figure 1) decreases the vulnerability of the resulting glycomimetic towards glycosidases, while the core structure and essential network of hydroxyl functionalities for enzyme recognition are retained. An important example is the 1-deoxynojirimycin (DNJ) family, for which DNJ itself is a competitive inhibitor of α-D-glucosidase (*K*_i_ = 8–25 μM),[10] while its derivatives miglustat (*N-n*Bu DNJ, Zavesa^®^) and miglitol (*N*-hydroxethyl DNJ, Glyset^®^ or Diastabol^®^) have already found therapeutic applications in Gaucher's disease [11] and type 2 (non-insulino-dependant *mellitus*) diabetes, [12–13] respectively. In the past decade, works have been targeted to carbasugars originally consisting of six-membered cyclitols, related to valiolamine, [14] voglibose, [15] valienamine, [16] and acarbose. [17] The last two compounds, marketed as Basen^®^ and Glucor^®^ or Precose^®^, respectively, are also actually used in the treatment of type 2 diabetes. All these compounds can have their amino moiety protonated, and the corresponding ammonium ions mimick the charge of the presumed transition states or intermediates of the enzymatic glycoside hydrolyses [18].

More recently, attention has been increasingly accorded to seven- and eight-membered ring systems [19–30] in order to study the effect of the enhanced flexibility and of the new spatial distribution displayed by these structures on their adaptability in the active site of the enzyme.

**Figure 2:**
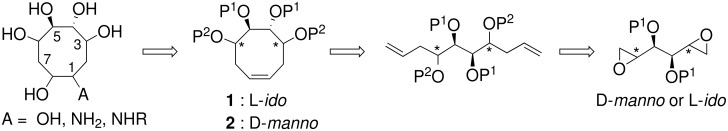
Retrosynthetic analysis.

As part of a program directed to the synthesis of potential glycosidases inhibitors, [31–32] we focused on the access to new eight-membered carbasugars (Figure 2, A = OH) and related aminocyclitols (A = NHR) from C_2_-symmetrical L-*ido*- or D-*manno*- cyclooctene, easily available by ring closing metathesis of 1,9-diene derived from L-*ido*- or D-*manno-*bis-epoxide [33]. Thus, synthetic potentialities of the newly created cyclic double bond were explored to reach hexa-substituted C8-glycomimetics.

## Results and discussion

**Scheme 1:**
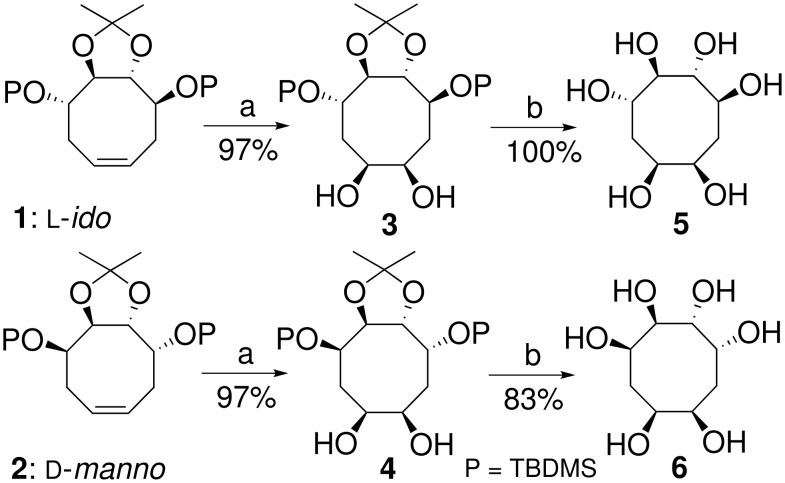
Reagents and conditions: (a) OsO_4_, NMO, *t*BuOH, rt; (b) TFA, H_2_O, rt.

From the C_2_-symmetrical L-*ido*- or D-*manno*- cyclooctene, **1** or **2**, to obtain the C8 hexa-substitued carbasugars a straightfoward approach seemed to be a dihydroxylation, whereas to obtain the corresponding aminocyclitols it could be an epoxidation followed by the nucleophilic opening of the epoxide moiety by a primary amine or another nitrogen nucleophile. Accordingly (Scheme 1), treatment of the fully *O*-protected L-*ido*-cyclooctene **1** with a 5 mol% aqueous solution of osmium(IV) tetroxide [34] in acetone in the presence of *N*-methylmorpholine oxide and *tert*-butanol cleanly led to the expected *cis*-diol **3** in 97% yield. In analogous manner, the D-*manno*-cyclooctene **2** gave the corresponding *cis*-diol **4** in 97% yield. In each case, the *cis*-diol **3** or **4** has been isolated as a single stereoisomer because of the C_2_-axis of symmetry displayed by the L-*ido* or D-*manno*-cyclooctenes **1** and **2**. Then, simultaneous acidic hydrolysis of all the *O*-protective groups of **3** and **4** furnished the C8 hexa-substitued carbasugars **5** and **6** (80 to 97% overall yield for the two steps).

**Scheme 2:**
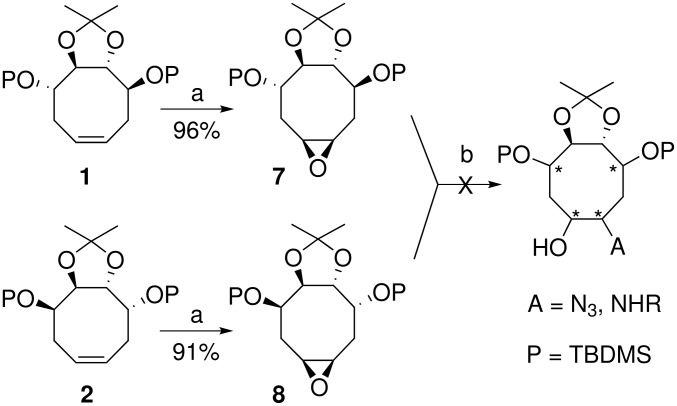
Reagents and conditions: (a) *m*CPBA, CH_2_Cl_2_, NaHCO_3_, rt; (b) see text.

Now, to reach the corresponding aminocyclitols, we turned to the epoxidation [35] of the cyclooctenes **1** and **2** (Scheme 2). Thus, treatment of **1** and **2** with *meta*-chloroperbenzoic acid in the presence of sodium hydrogen carbonate afforded the epoxides **7** and **8** in 91–96% yield. As precedently, because of the C_2_-axis of symmetry displayed by the L-*ido* or D-*manno*-cyclooctenes **1** and **2** the *cis*-epoxide **7** or **8** has been isolated as a single stereoisomer. However, all attempts to open the epoxide ring involving various nucleophiles, sodium azide, benzylamine, *n*-butylamine, or serinol in different experimental conditions, protic or aprotic solvent, presence or absence of a Lewis acid catalyst such as ytterbium triflate, revealed unsuccessful, only leading to recover the starting material.

**Scheme 3:**
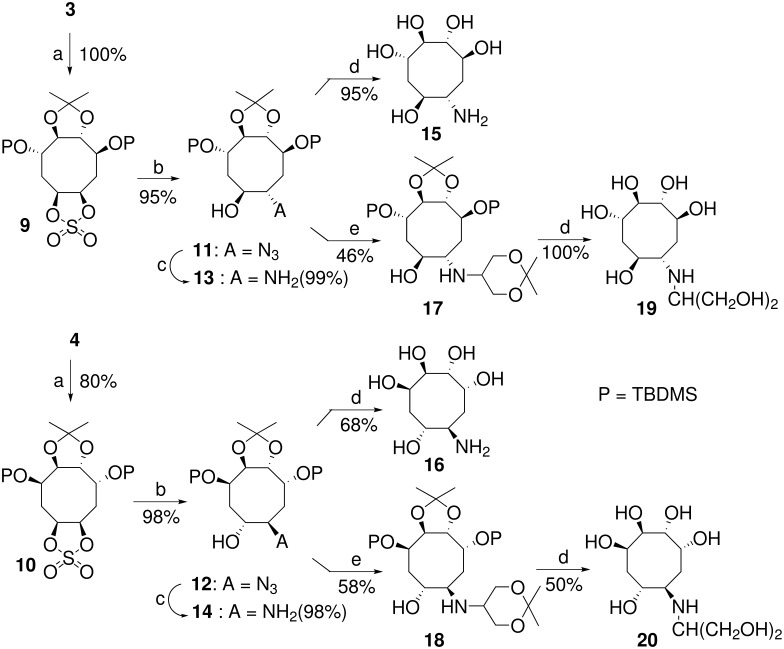
Reagents and conditions: (a) *i* : SOCl_2_, Et_3_N, CH_2_Cl_2_, 0°C; *ii* : RuCl_3_, NaIO_4_, CCl_4_, CH_3_CN, 0°C to rt; (b) NaN_3_, DMF, 80°C; (c) H_2_, Pd black, EtOAc; (d) *i:* TFA, H_2_O, rt; *ii:* Dowex-50WX8 H^+^ resin, 1% NH_4_OH; (e) Ti(O*i*Pr)_4_, OC(CH_2_O)_2_CMe_2_, CH_2_Cl_2_ then NaBH_3_CN, EtOH.

To overcome this difficulty, we turned to a more electrophilic sulfate moiety [36] (Scheme 3). Thus, treatment of the *cis*-diols **3** and **4** with thionyl chloride in the presence of triethylamine followed by subsequent oxidation with sodium periodate in the presence of ruthenium trichloride gave the cyclic sulfates **9** and **10** in 80–100% yield. Nucleophilic opening of these sulfates by sodium azide in DMF at 80°C, [37] followed by acidic hydrolysis of the resulting acyclic sulfate ester cleanly afforded the corresponding azido-alcohols **11** and **12**, isolated as single stereoisomers in excellent yield (95–98%). No other isomer of **11** or **12** was detected by NMR analysis, indicating that the ring-opening reaction is highly regioselective and results in the *anti* addition of the azido group on the opposite side of the bulky TBDMS group in β-position. However, it has to be pointed out that more hindered nucleophiles, such as primary amines, revealed unable to open the cyclic sulfate **9** or **10**.

The absolute configurations of **11** and **12** were established by NMR studies. ^1^H signals were assigned (Table 1) using 2D-COSY and 2D-TOCSY experiments starting from hydroxyl group at C8-position.

**Table 1:** Selected ^1^H NMR data for compound **11** in CDCl_3_ at 500 MHz.

Proton	δ^1^H (ppm)	^2^*J*,^3^*J* (Hz)

1	3.73	^3^*J*_1-2a_ = 2.2 ^3^*J*_1-2b_ = 9.0 ^3^*J*_1-8_ = 9.5
2a (*pro*S)	2.06	^2^*J*_2a-2b_ = -15.4 ^3^*J*_2a-3_ = 6.5
2b (*pro*R)	1.90	^3^*J*_2b-3_ = 1.5
3	4.04	^3^*J*_3-4_ = 4.2
4	3.50	^3^*J*_4-5_ = 9.5
5	3.59	^3^*J*_5-6_ = 6.3
6	3.94	^3^*J*_6-7a_ = 1.8 ^3^*J*_6-7b_ = 5.5
7a (*pro*R)	2.06	^2^*J*_7a-7b_ = -15.4 ^3^*J*_7a-8_ = 8.8
7b (*pro*S)	1.95	^3^*J*_7b-8_ = 1.5
8	3.73	
8-OH	2.39	

**Figure 3:**
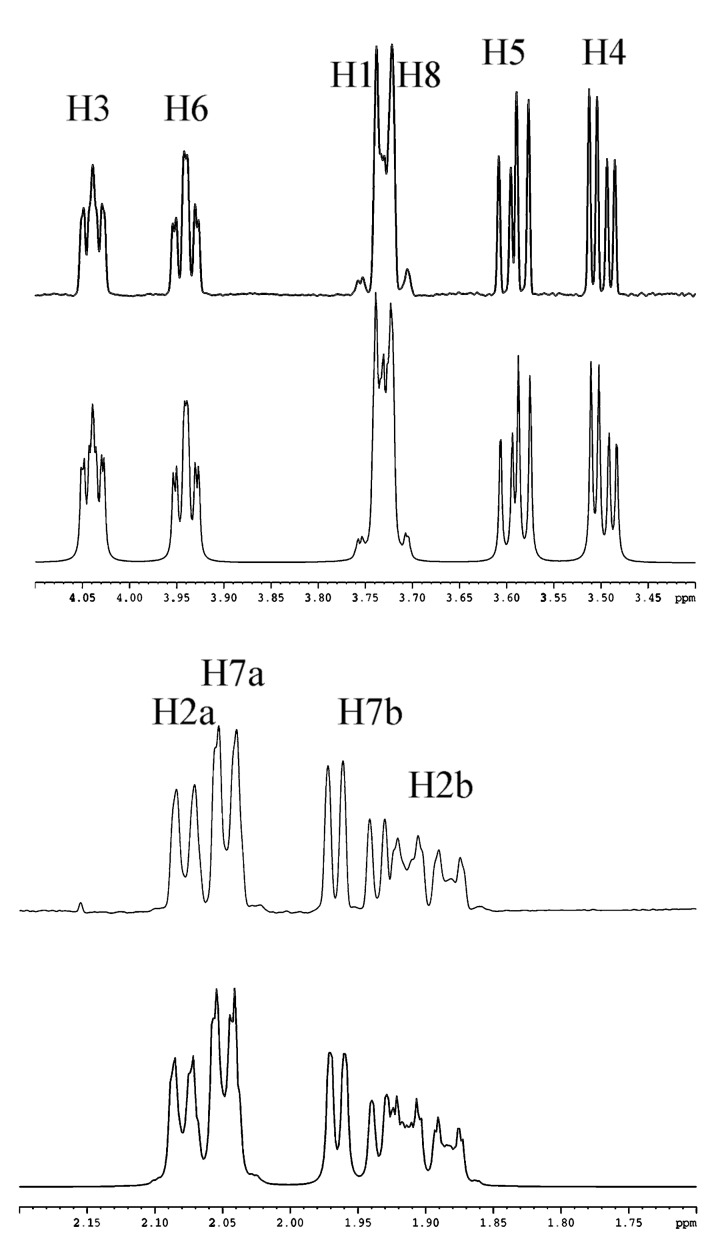
1D proton NMR spectra of the C8 ring in compound **11** (upper) and the simulated signals (down) on the basis of the chemical shifts and coupling constants summarized in Table 1.

**Figure 4:**
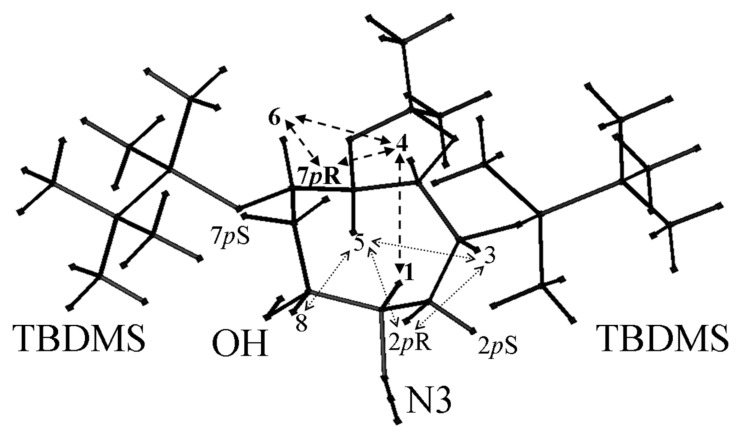
Schematic representation of the NOEs (indicated with arrows) found to deduce the structure of **11**. Bold arrows show the NOEs found between the protons (in bold) of the upper face of the C8 ring. Prochiral ^1^H are labelled *p*R or *p*S.

The determination of all the coupling constants and particularly of the ^3^*J*_1,8_ was not possible by homodecoupling experiments. Thus, numerical simulation was used for an in depth study of the complex coupling patterns to set the parameters in complete analogy with regard to the experimental spectra. These ^3^*J*_1H,1H_ coupling constants, determined by simulation (Figure 3) of 1D spectra with NMR-SIM started from XWIN-NMR software (Bruker), are gathered in Table 1. These values allowed us to restrain the number of conformations for the eight-membered ring. For example, in compound **11**, the large ^3^*J*_1H,1H_ coupling constants found between H1 and H8, H1 and H2 (*pro*R), H8 and H7 (*pro*R), H4 and H5, are in agreement with protons in a pseudo-axial position. On the other hand, the small ^3^*J*_1H,1H_ coupling constants found between H1 and H2 (*pro*S), H8 and H7 (*pro*S) indicate a pseudo-equatorial position of protons H2 (*pro*S) and H7 (*pro*S). NOE measurements and finally Molecular Dynamic calculations using Insight II software (Biosym Technologies, San Diego, CA) allowed to deduce the structure of **11** (Figure 4). Prochiral H2 (*pro*S) and H7 (*pro*S) protons displayed strong NOEs with the TBDMS groups, respectively in positions 3 and 6, indicating they are pointing away from the C8 ring in a pseudo-equatorial position. Hence, the protons H7 (proR), H6, H4 are close together and represent one face (upper) of the C8 ring, while H2 (proR), H5, H3 represent the other face. Then, in compound **11,** the strong NOEs H1-H4 and H8-H5 indicate that H1 and H8 are in opposite side in an *anti* configuration with pseudo-axial positions. A similar approach was applied for the structural determination of **12**.

**Figure 5:**
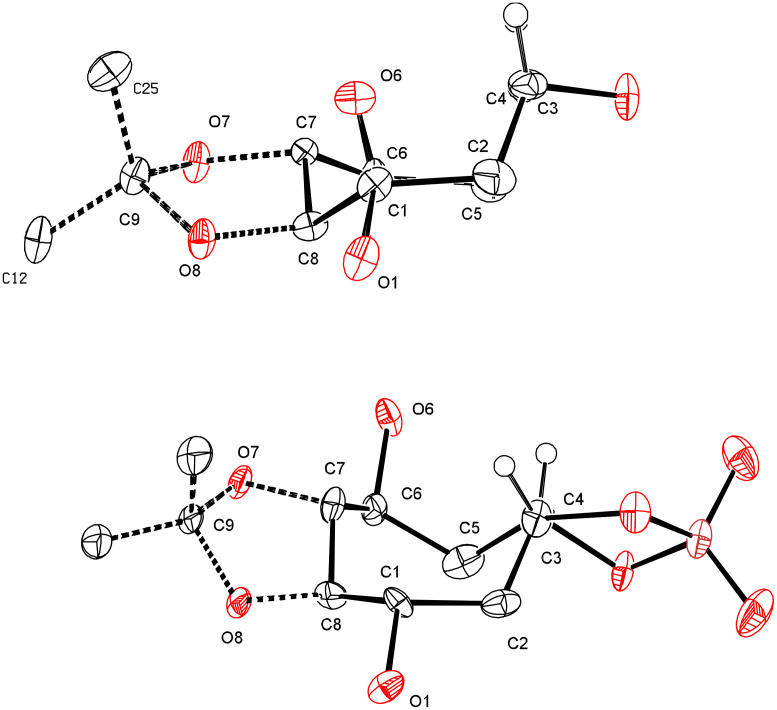
X-ray structure of epoxide **7** (upper) and sulfate **9** (down) solved using SHELXS and anisotropically refined using SHELXL programs [38].

The low reactivity of the epoxide function of **7** could be explained by the steric hindrance of the *tert*-butyldimethylsilyloxy groups in β-positions as confirmed by the X-ray crystallography (Figure 5). Furthermore, it seems that the C8-carbacycle of the tricyclic system [5-8-3] adopts a twist-boat-chair conformation. Whereas that of the sulfate **9**, which crystallizes as a dimer, constituted by a tricyclic system [5-8-5] adopts a more flexible boat-chair conformation, thus allowing its opening by a linear nucleophile (azide anion), but not by a more hindered nucleophile (primary amine).

With the key enantiomerically pure azido-alcohol **11** and **12** in hands, we next turned to the obtention of C8-aminocyclitols. Thus, reduction of the azido group of **11** by dihydrogen in the presence of palladium black in ethyl acetate (Scheme 3) afforded the amino-alcohol **13** which could be submitted to acidic hydrolysis of the *O*-protective groups to give, after purification by ion-exchange chromatography, the targeted aminocyclitol **15** [20] (95% overall yield from **11**). Alternatively, to obtain an analog of voglibose, the amine function of **13** could be alkylated *via* a reductive amination [39] with a dihydroxyacetone derivative. Thus, treatment of the amine **13** by the commercially available 2,2-dimethyl-1,3-dioxan-5-one in the presence of titanium(IV) tetra-isopropoxide followed by the cyanoborohydride reduction of the imine intermediate gave the expected *N*-alkylated aminocyclitol **17** (46% overall yield from **11**). Then, simultaneous acidic hydrolysis of all protective groups led to the C8-voglibose mimetic **19** after purification by ion-exchange chromatography. The same sequence of reactions was uneventfully applied to the azido-alcohol **12** to afford the aminocyclitols **16** and **20**.

The new C8-carbasugars **5** and **6** and C8-aminocyclitols **15**, **16**, **19** and **20** have been assayed for their inhibitory activity towards 24 commercially available glycosidases [40–41]. They did not inhibit the following enzymes at 1 mM concentration and optimal pH : α-D-glucosidases (maltase) from yeast and rice, β-D-glucosidase from *caldocellum saccharolyticum*, α-L-fucosidases from bovine epididymis and human placenta, α-D-galactosidases from coffee beans and *Escherichia coli*, β-D-galactosidases from *Escherichia coli*, bovine liver, *Aspergillus niger* and *Aspergillus orizae*, α-*N*-acetylgalactosaminidase from chicken liver, β-*N*-acetylglucosaminidases from Jack bean, bovine epididymis A and bovine epididymis B, α-D-mannosidase from almonds, β-D-mannosidase from *Helix pomatia*, and β-xylosidase from *Aspergillus niger*. For other enzymes: α-D-glucosidase from *Bacillus stearothermophilus*, amyloglucosidase from *Aspergillus niger* and *Rhizopus* mold, β-D-glucosidase from almonds, α-L-fucosidase from bovine kidney, and α-D-mannosidase from Jack beans the results are shown in Table 2. Each of these new compounds revealed weak inhibitor of the tested enzymes with a percentage of inhibition not over than 30%. These results show that the enhanced flexibility displayed by C8-glycomimetics does not seem to be correlated with an increase in observed activity. Thus, for example we had previously shown that the corresponding C7-voglibose mimic exhibited interesting activity towards amyloglucosidases from *Aspergillus niger* and *Rhizopus* mold (35 and 18 μM respectively, unpublished results). Furthermore, even if data concerning biological activity of C8-glycomimetics are seldom, the reported activities are often weak [24–30].

**Table 2:** Inhibitory activities for C8-carbasugars **5–6**, and for C8-aminocyclitols **15**, **16**, **19** and **20**. Percentage of inhibitions at 1 mM.

Enzyme^a^	**5**	**6**	**15**	**16**	**19**	**20**

α-D-Glucosidase						
- *Aspergillus niger*	n.i.^b^	n.i.^b^	28%	n.i.^b^	27%	29%
- *Rhizopus* mold	20%	21%	23%	20%	28%	27%
- *Bac. stearotherm.^b^*	9%	n.i.^b^	n.i.^b^	5%	n.i.^b^	6%
β-D-Glucosidase	n.i.^b^	n.i.^b^	7%	11%	6%	19%
α-D-Mannosidase	5%	n.i.^b^	16%	8%	10%	8%
α-L-Fucosidase	14%	13%	n.i.^b^	n.i.^b^	n.i.^b^	n.i.^b^

^a^See text; ^b^No inhibition detected.

In summary, utilizing the readily available polyhydroxylated L-ido or D-manno-cyclooctenes, coming from ring closing metathesis of C2-symmetrical 1,9-dienes, we have accomplished the synthesis of a range of new hexa-substituted C8-glycomimetics in enantiopure form. Transformation of the cyclic double bond involved *syn*-dihydroxylation, then introduction of an azido group by opening of a cyclic sulfate followed by reduction and eventual alkylation of the resulting amine.

According to this strategy and to the nature of the ketones involved in the final reductive amination, various aminocyclitols could be synthesized. Thus, in this study two carbasugars and four aminocyclitols were obtained. Biological evaluation of these compounds towards 24 commercially available glycosidases have been carried out. For these C8-glycomimetics, weak activities were observed, which can probably be explained by a too high conformational flexibility of such structures.

## Experimental

See Supporting Information File 1.

## Supporting Information

File 1 Additional information.
