# Supplementary material for: Synthesis and glycosidase inhibitory activity of new hexa-substituted C8-glycomimetics
Source: Beilstein J Org Chem. 2005 Oct 7;1:12. doi: 10.1186/1860-5397-1-12 (PMC1399460; doi:10.1186/1860-5397-1-12)
Supplement: File 1 — Additional information. [file Beilstein_J_Org_Chem-01-12-s001.doc]

# Experimental

^1^H NMR (250 MHz) and ^13^C NMR (63 MHz) spectra were recorded on a Bruker AM250 in CDCl_3_ (unless indicated). Chemical shifts (δ) are reported in ppm and coupling constants are given in Hz. Optical rotations were measured on a Perkin-Elmer 241C polarimeter with sodium (589 nm) or mercury (365 nm) lamp. Mass spectra, chemical ionization (CI), and high resolution (HRMS) were recorded by the Service de Spectrométrie de Masse, Ecole Normale Supérieure, Paris. All reactions were carried out under an argon atmosphere, and were monitored by thin-layer chromatography with Merck 60F-254 precoated silica (0.2 mm) on glass. Flash chromatography was performed with Merck Kieselgel 60 (0.2-0.5 mm); the solvent system were given v/v. spectroscopic; (^1^H and ^13^C NMR, MS) and/or analytical data were obtained using chromatographically homogeneous samples.

**1- General procedure for dihydroxylation**

To a solution of cyclooctene (1.40 mmol) in acetone (20 mL) and *tert*-butanol (8 mL) was added *N*-methylmorpholine oxide (2.8 mmol, 2 equiv.). After stirring at 20°C for 20 min., osmium tetroxide (0.5 M aqueous solution, 0.056 mmol, 4 mol%) was added. After stirring at 20°C for 4h, sodium sulfite (3.50 mmol, 2.5 equiv.) was added. The mixture was filtered and the filtrate was concentrated in vacuo. Flash chromatography of the crude led to the corresponding pure *syn-*diol.

**1.1- (1*S*,2*S*,3*S*,4*S*,6*R*,7*S*)-1,4-Di-*O*-*tert*-butyldimethylsilyl-2,3-*O*-methylethylidene-cyclooctane-1,2,3,4,6,7-hexol (3).**

Isolated yield: 97%; *Rf* 0.3 (cyclohexane/EtOAc 9:1); mp 110°C; [α]_D_= +35 (*c*1.0, CH_2_Cl_2_); ^1^H NMR *δ* 4.06 (dddd, 1H, *J*_8,1_= 3.5 Hz, *J*_8,7a_= 4.0 Hz, *J*_8,7b_= 10.4 Hz, *J*_8,OH_= 4.1 Hz, H_8_), 3.95-3.80 (m, 3H, H_1,3,6_), 3.75 (dd, 1H, *J*_4,3_= 8.0 Hz, *J*_4,5_= 8.8 Hz, H_4_), 3.65 (dd, 1H, *J*_5,4_= 8.8 Hz, *J*_5,6_= 7.0 Hz, H_5_), 2.29 (d, 1H, *J*_OH,8_= 4.1 Hz, OH_8_), 2.18 (ddd, 1H, *J*_2a,1_= *J*_2a,3_= 9.0 Hz, *J*_2a,2b_= 15.3 Hz, H_2a_), 1.98 (ddd, 1H, *J*_7a,6_= 5.8 Hz, *J*_7a,7b_= 14.8 Hz, *J*_7a,8_= 4.0 Hz, H_7a_), 1.88-1.76 (m, 1H, H_7b_), 1.70 (ddd, 1H, *J*_2b,1_= 4.2 Hz, *J*_2b,2a_= 15.3 Hz, *J*_2b,3_= 2.0 Hz, H_2b_), 1.32, 1.31 (2s, 6H, CH_3_), 0.88, 0.87 (2s, 18H, Si*t*Bu), 0.09, 0.07 (2s, 12H, SiMe_2_); ^13^C NMR *δ* 108.0 (CMe_2_), 82.1 (C_5_), 81.5 (C_4_), 75.2 (C_3_), 72.8 (C_6_), 69.3 (C_1_), 68.5 (C_8_), 36.4 (C_2,7_), 27.0, 26.7 (CMe_2_), 25.8 (SiC(CH_3_)_3_), 18.1 (SiC(CH_3_)_3_), -4.3, -4.5, -5.1 (SiMe_2_); Anal. calcd for C_23_H_48_O_6_Si_2_: C, 57.94; H, 10.15; found C, 58.00; H, 10.15.

**1.2- (1*R*,2*S*,3*S*,4*R*,6*R*,7*S*)-1,4-Di-*O*-*tert*-butyldimethylsilyl-2,3-*O*-methylethylidene-cyclooctane-1,2,3,4,6,7-hexol (4).**

Isolated yield: 97%; *Rf* 0.25 (cyclohexane/EtOAc 8:2); mp 120°C; [α]_D_= -29 (*c*1.0, CH_2_Cl_2_); ^1^H NMR *δ* 4.37 (ddd, 1H, *J*_8,1_= 1.4 Hz, *J*_8,7a_= 7.9 Hz, *J*_8,7b_= 2.7 Hz, H_8_), 4.29 (ddd, 1H, *J*_1,2a_= 1.0 Hz, *J*_1,2b_= 8.3 Hz, *J*_1,8_= 1.4 Hz, H_1_), 4.11 (ddd, 1H, *J*_6,5_= 1.4 Hz, *J*_6,7a_= 7.4 Hz, *J*_6,7b_= 2.4 Hz, H_6_), 4.04 (dd, 1H, *J*_4,3_= 1.4 Hz, *J*_4,5_= 8.6 Hz, H_4_), 4.02-3.91 (m, 1H, H_3_), 3.97 (dd, 1H, *J*_5,4_= 8.6 Hz, *J*_5,6_= 1.4 Hz, H_5_), 2.30 (ddd, 1H, *J*_7a,6_= 7.4 Hz, *J*_7a,7b_= 15.5 Hz, *J*_7a,8_= 7.9 Hz, H_7a_), 2.15 (ddd, 1H, *J*_2a,1_= 1.0 Hz, *J*_2a,2b_= 15.0 Hz, *J*_2a,3_= 9.9 Hz, H_2a_), 1.99 (ddd, 1H, *J*_2b,1_= 8.3 Hz, *J*_2b,2a_= 15.0 Hz, *J*_2b,3_= 2.2 Hz, H_2b_), 1.77 (ddd, 1H, *J*_7b,6_= 2.4 Hz, *J*_7b,7a_= 15.5 Hz, *J*_7b,8_= 2.7 Hz, H_7b_), 1.34 (s, 6H, CH_3_), 0.92 (s, 18H, Si*t*Bu), 0.15, 0.12, 0.10 (3s, 12H, SiMe_2_); ^13^C NMR *δ* 106.7 (CMe_2_), 78.8 (C_4,5_), 76.8 (C_3,6_), 69.8 (C_8_), 67.0 (C_1_), 35.9 (C_2,7_), 27.2 (CMe_2_), 26.0, 25.9 (SiC(CH_3_)_3_), 18.1, 18.0 (SiC(CH_3_)_3_), -4.4, -4.6, -4.8, -4.9 (SiMe_2_); Anal. calcd for C_23_H_48_O_6_Si_2_: C, 57.94; H, 10.15; found C, 57.88; H, 10.14.

**2- Deprotection of the cyclooctanols**

The substrate (0.33 mmol) was stirred in a 9:1 solution of trifluoroacetic acid/H_2_O at 20°C for 15h. After concentration in vacuo, the resulting oily residue was triturated in diethyl ether and the supernatant was discarded to afford a brownish solid which was then purified by reverse phase chromatography (Sep-Pak® Cartridges, H_2_O/MeOH 1:1 to 1:5).

**2.1- (1*S*,2*R*,3*R*,4*S*,6*R*,7*S*)-cyclooctane-1,2,3,4,6,7-hexol (5).**

Isolated yield: 100%; [α]_D_ +2 (*c*1.0, D_2_O); ^1^H NMR (D_2_O) *δ* 4.08 (ddd, 1H, *J*_8,1_= 3.2 Hz, *J*_8,7a_= 9.9 Hz, *J*_8,7b_= 4.8 Hz, H_8_), 3.96 (ddd, 1H, *J*_1,2a_= 9.2 Hz, *J*_1,2b_= 3.0 Hz, *J*_1,8_= 3.2 Hz, H_1_), 3.90 (ddd, 1H, *J*_6,5_= 8.7 Hz, *J*_6,7a_= 3.4 Hz, *J*_6, 7b_= 8.0 Hz, H_6_), 3.75 (ddd, 1H, *J*_3,2a_= 10.0 Hz, *J*_3,2b_= 3.0 Hz, *J*_3,4_= 8.7 Hz, H_3_), 3.53 (dd, 1H, *J*_5,4_= *J*_5,6_= 8.7 Hz, H_5_), 3.41 (dd, 1H, *J*_4,3_= *J*_4,5_= 8.7 Hz, H_4_), 2.18-1.95 (m, 1H, H_2a_), 2.12 (ddd, 1H, *J*_7a,6_= 3.4 Hz, *J*_7a,7b_= 15.0 Hz, *J*_7a,8_= 9.9 Hz, H_7a_), 1.99 (dddd, 1H, *J*_7b,6_= 8.0 Hz, *J*_7b, 7a_ = 15.0 Hz, *J*_7b,8_= 4.8 Hz, H_7b_), 1.88 (ddd, 1H, *J*_2b,1_= 3.0 Hz, *J*_2b, 2a_= 15.1 Hz, *J*_2b,3_= 3.0 Hz, H_2b_); ^13^C NMR (D_2_O) *δ* 77.5 (C_4_), 76.3 (C_5_), 75.0 (C_3_), 73.1 (C_6_), 71.4 (C_8_), 70.4 (C_1_), 38.3 (C_2_), 37.7 (C_7_); HRMS for C_8_H_17_O_6_ (M^+^+1): calcd 209.1025; found 209.1026.

**2.2- (1*R*,2*R*,3*R*,4*R*,6*R*,7*S*)-cyclooctane-1,2,3,4,6,7-hexol (6).**

Isolated yield: 83%; [α]_D_ -11 (*c*1.0, D_2_O); ^1^H NMR (D_2_O) *δ* 4.34 (ddd, 1H, *J*_3,2a_= 8.4 Hz, *J*_3,2b_= 2.2 Hz, *J*_3,4_= 2.5 Hz, H_3_), 4.20-4.08 (m, 2H, H_1,6_), 3.95 (dd, 1H, *J*_4,3_= 2.5 Hz, *J*_4,5_= 8.2 Hz, H_4_), 3.89 (ddd, 1H, *J*_8,1_= *J*_8,7b_= 2.4 Hz, *J*_8,7a_= 10.1 Hz, H_8_), 3.68 (dd, 1H, *J*_5,4_= 8.2 Hz, *J*_5,6_= 2.1 Hz, H_5_), 2.28-2.02 (m, 1H, H_7a_), 2.09 (ddd, 1H, *J*_2a,1_= 2.0 Hz, *J*_2a,2b_= 15.6 Hz, *J*_2a,3_= 8.4 Hz, H_2a_), 1.93 (ddd, 1H, *J*_2b,1_= 7.4 Hz, *J*_2b, 2a_= 15.6 Hz, *J*_2b,3_= 2.2 Hz, H_2b_), 1.94-1.81 (m, 1H, H_7b_); ^13^C NMR (D_2_O) *δ* 76.5 (C_4_), 76.1 (C_5_), 72.3 (C_8_), 71.5 (C_1_), 71.0 (C_6_), 66.8 (C_3_), 37.1 (C_7_), 36.9 (C_2_); HRMS for C_8_H_20_NO_6_ (M^+^+18): calcd 226.1291; found 226.1288.

**3- Formation of epoxides**

To a solution of cyclooctene (0.45 mmol) in CH_2_Cl_2_ (4 mL) were added sodium hydrogenocarbonate (1.04 mmol, 2.3 equiv.) and *meta*-chloroperbenzoic acid (purity 57 to 86 %, 0.81 mmol, 1.8 equiv.). After stirring at 20°C for 2h, a saturated sodium sulfite aqueous solution was added. After extraction of the mixture with CH_2_Cl_2_, the combined organic layers were washed with a 20% sodium sulfite aqueous solution, then with a 5% sodium hydrogenocarbonate aqueous solution, then with water, dried (MgSO_4_), filtered and concentrated in vacuo. Flash chromatography of the crude led to pure epoxide derivatives.

**3.1- (1*S*,2*S*,3*S*,4*S*,6*R*,7*S*)-6,7-Anhydro-1,4-di-*O*-*tert*-butyldimethylsilyl-2,3-*O*-methylethylidene-cyclooctane-1,2,3,4,6,7-hexol (7).**

Isolated yield: 96%; *Rf* 0.3 (cyclohexane/EtOAc 97:3); mp 70-71°C; [α]_D_= +31 (*c*1.0, CH_2_Cl_2_); ^1^H NMR *δ* 3.91-3.80 (m, 2H, H_4,5_), 3.74 (ddd, 1H, *J*_3,2a_= 2.7 Hz, *J*_3,2b_=8.6 Hz, *J*_3,4_= 11.4 Hz, H_3_), 3.54 (ddd, 1H, *J*_6,5_= 8.1 Hz, *J*_6,7a_= 3.8 Hz, *J*_6,7b_= 4.3 Hz, H_6_), 3.24 (ddd, 1H, *J*_8,1_= 4.2 Hz, *J*_8,7a_= 3.3 Hz, *J*_8,7b_= 11.0 Hz, H_8_), 2.91 (ddd, 1H, *J*_1,2a_= 4.7 Hz, *J*_1,2b_= 9.8 Hz, *J*_1,8_= 4.2 Hz, H_1_), 2.33 (ddd, 1H, *J*_2a,1_= 4.7 Hz, *J*_2a,2b_= 14.3 Hz, *J*_2a,3_= 2.7 Hz, H_2a_), 2.17 (ddd, 1H, *J*_7a,6_= 3.8 Hz, *J*_7a,8_= 3.3 Hz, *J*_7a,7b_= 14.1 Hz, H_7_), 1.60-1.30 (m, 2H, H_2b,7b_), 1.36, 1.32 (2s, 6H, CH_3_), 0.88, 0.87 (2s, 18H, Si*t*Bu), 0.09, 0.08 (2s, 12H, SiMe_2_); ^13^C NMR *δ* 107.8 (CMe_2_), 82.0, 81.5 (C_4,5_), 73.8, 72.5 (C_3,6_), 52.1, 51.3 (C_1,8_), 35.6, 31.9 (C_2,7_), 27.0, 26.6 (CMe_2_), 25.8, 25.7 (SiC(CH_3_)_3_), 18.3, 18.1 (SiC(CH_3_)_3_), -4.3, -4.4, -5.0, -5.2 (SiMe_2_); HRMS for C_23_H_47_O_5_Si_2_ (M^+^+1): calcd 459.2962; found 459.2959.

**3.2- (1*R*,2*S*,3*S*,4*R*,6*R*,7*S*)-6,7-Anhydro-1,4-di-*O*-*tert*-butyldimethylsilyl-2,3-*O*-methylethylidene-cyclooctane-1,2,3,4,6,7-hexol (8).**

Isolated yield: 91%; *Rf* 0.3 (cyclohexane/CH_2_Cl_2_ 8:4); mp 115-117°C; [α]_D_= -61 (*c*1.0, CH_2_Cl_2_); ^1^H NMR *δ* 4.31 (dd, 1H, *J*_4,3_= 3.1 Hz, *J*_4,5_= 7.7 Hz, H_4_), 4.28-4.20 (m, 1H, H_6_), 4.19 (ddd, 1H, *J*_3,2a_= 7.2 Hz, *J*_3,2b_= 2.9 Hz, *J*_3,4_= 3.1 Hz, H_3_), 3.99 (dd, 1H, *J*_5,4_= 7.7 Hz, *J*_5,6_= 1.8 Hz, H_5_), 3.14 (ddd, 1H, *J*_1,2a_= 4.5 Hz, *J*_1,2b_= 9.3 Hz, *J*_1,8_= 3.9 Hz, H_1_), 2.85 (ddd, 1H, *J*_8,1_= 3.9 Hz, *J*_8,7a_= 3.9 Hz, *J*_8,7b_= 11.3 Hz, H_8_), 2.55-2.40 (m, 2H, H_2a,7a_), 1.39, 1.37 (2s, 6H, CH_3_), 1.25 (ddd, 1H, *J*_2b,1_= 9.3 Hz, *J*_2b,2a_= 14.6 Hz, *J*_2b,3_= 2.9 Hz, H_2b_), 1.10 (ddd, 1H, *J*_7b,6_= 2.4 Hz, *J*_7b,7a_= 13.6 Hz, *J*_7b,8_= 11.3 Hz, H_7b_), 0.90, 0.88 (2s, 18H, Si*t*Bu), 0.09, 0.07 (2s, 12H, SiMe_2_); ^13^C NMR *δ* 108.8 (CMe_2_), 78.6 (C_5_), 76.9 (C_4_), 68.4, 66.7 (C_3,6_), 52.7 (C_1_), 51.5 (C_8_), 33.7, 31.0 (C_2,7_), 27.7, 27.5 (CMe_2_), 26.0 (SiC(CH_3_)_3_), 18.2 (SiC(CH_3_)_3_), -4.1, -4.8, -5.1 (SiMe_2_); HRMS for C_23_H_47_O_5_Si_2_ (M^+^+1): calcd 459.2962; found 459.2958.

**4- Formation of cyclic sulfates**

To a solution of diol (0.84 mmol) in CH_2_Cl_2_ (8 mL) at 0°C were added triethylamine (3.36 mmol, 4 equiv.) and thionyle chloride (2.94 mmol, 3.5 equiv.). The mixture was stirred at 0°C for 10 min, then diluted with diethyl ether (12 mL) and washed with cold water (12 mL). The organic layer was then dried (MgSO_4_), filtered and concentrated in vacuo. The residue was dissolved in CCl_4_ (8 mL) and acetonitrile (8 mL). The solution was cooled to 0°C and cold water (12 mL) was added. Ruthenium chloride (0.034 mmol, 4 mol%) and sodium periodate (1.26 mmol, 1.5 equiv.) were added. After stirring at 20°C for 1h, diethyl ether (16 mL) was added and the two layers were separated. The aqueous layer was extracted three times with diethyl ether and the combined organic layers were dried (MgSO_4_), filtered and concentrated in vacuo. Flash chromatography of the crude led to pure cyclic sulfate derivatives.

**4.1- (1*S*,2*S*,3*S*,4*S*,6*R*,7*S*)-1,4-Di-*O*-*tert*-butyldimethylsilyl-2,3-*O*-methylethylidene-6,7-*O*-sulfonyl-cyclooctane-1,2,3,4,6,7-hexol (9).**

Isolated yield: 100%; *Rf* 0.3 (cyclohexane/EtOAc 96:4); mp 110°C; [α]_D_= +72 (*c*1.0, CH_2_Cl_2_); ^1^H NMR *δ* 5.36 (ddd, 1H, *J*_8,1_= 5.6 Hz, *J*_8,7a_= 3.3 Hz, *J*_8,7b_= 11.4 Hz, H_8_), 4.89 (ddd, 1H, *J*_1,2a_= 11.4 Hz, *J*_1,8_= 5.6 Hz, H_1_), 3.89 (ddd, 1H, *J*_6,5_= 8.6 Hz, *J*_6,7a_= 4.4 Hz, *J*_6,7b_= 2.6 Hz, H_6_), 3.72 (ddd, 1H, *J*_3,2a_= 10.8 Hz, *J*_3,2b_= 4.4 Hz, *J*_3,4_= 8.4 Hz, H_3_), 3.60 (dd, 1H, *J*_5,4_= 8.5 Hz, *J*_5,6_= 8.6 Hz, H_5_), 3.24 (dd, 1H, *J*_4,3_= 8.4 Hz, *J*_4,5_= 8.5 Hz, H_4_), 2.42 (ddd, 1H, *J*_2a,1_= 11.4 Hz, *J*_2a,2b_= 15.2 Hz, *J*_2a,3_= 10.8 Hz, H_2a_), 2.28 (ddd, 1H, *J*_7a,6_= 4.4 Hz, *J*_7a,7b_= 14.6 Hz, *J*_7a,8_= 3.3 Hz, H_7a_), 2.19–2.02 (m, 2H, H_2b,7b_), 1.35, 1.33 (2s, 6H, CH_3_), 0.88, 0.87 (2s, 18H, Si*t*Bu), 0.08, 0.07, 0.06, 0.04 (4s, 12H, SiMe_2_); ^13^C NMR *δ* 109.2 (CMe_2_), 82.1, 81.0 (C_1,8_), 80.7, 79.9 (C_4,5_), 73.7 (C_3_), 71.5 (C_6_), 35.7, 32.5 (C_2,7_), 27.0, 26.6 (CMe_2_), 25.7 (SiC(CH_3_)_3_), 18.2, 18.0 (SiC(CH_3_)_3_), -4.3, -4.4, -5.0, -5.2 (SiMe_2_); HRMS for C_23_H_47_O_8_SSi_2_ (M^+^+1): calcd 539.2530; found 539.2536.

**4.2- (1*R*,2*S*,3*S*,4*R*,6*R*,7*S*)-1,4-Di-*O*-*tert*-butyldimethylsilyl-2,3-*O*-methylethylidene-6,7-*O*-sulfonyl-cyclooctane-1,2,3,4,6,7-hexol (10).**

Isolated yield: 80%; *Rf* 0.3 (cyclohexane/EtOAc 95:5); mp 180-181°C; [α]_D_= -16 (*c*1.0, CH_2_Cl_2_); ^1^H NMR *δ* 5.34 (ddd, 1H, *J*_1,2a_= 2.6 Hz, *J*_1,2b_= 9.1 Hz, *J*_1,8_= 6.2 Hz, H_1_), 5.00 (ddd, 1H, *J*_8,1_= 6.2 Hz, *J*_8,7a_= 2.3 Hz, *J*_8,7b_= 12.2 Hz, H_8_), 4.30 (ddd, 1H, *J*_3,2a_= 6.4 Hz, *J*_3,2b_= 2.2 Hz, *J*_3,4_= 2.3 Hz, H_3_), 4.10 (ddd, 1H, *J*_6,5_= 3.1 Hz, *J*_6,7a_= 6.8 Hz, *J*_6,7b_= 10.8 Hz, H_6_), 4.02 (dd, 1H, *J*_4,3_= 2.3 Hz, *J*_4,5_= 8.0 Hz, H_4_), 3.68 (dd, 1H, *J*_5,4_= 8.0 Hz, *J*_5,6_= 3.1 Hz, H_5_), 2.41 (ddd, 1H, *J*_7a,6_= 6.8 Hz, *J*_7a,7b_= 14.5 Hz, *J*_7a,8_= 2.3 Hz, H_7a_), 2.33–2.17 (m, 2H, H_2a,2b_), 1.93 (ddd, 1H, *J*_7b,6_= 10.8 Hz, *J*_7b,7a_= 14.5 Hz, *J*_7b,8_= 12.2 Hz, H_7b_), 1.37, 1.36 (2s, 6H, CH_3_), 0.88, 0.87 (2s, 18H, Si*t*Bu), 0.13, 0.11, 0.10, 0.08 (4s, 12H, SiMe_2_); ^13^C NMR *δ* 110.0 (CMe_2_), 80.4 (C_1_), 79.2 (C_8_), 77.3 (C_4_), 75.4 (C_5_), 65.8 (C_3_), 65.4 (C_6_), 33.9 (C_2_), 32.6 (C_7_), 27.7, 27.4 (CMe_2_), 26.0 (SiC(CH_3_)_3_), 18.1 (SiC(CH_3_)_3_), -5.3 (SiMe_2_); Anal. calcd for C_23_H_46_O_8_SSi_2_: C, 51.27; H, 8.60; found C, 51.19; H, 8.70.

**5- Cyclic sulfate opening**

To a solution of cyclic-sulfate (1.49 mmol) in DMF (2 mL) was added sodium azide (7.43 mmol, 5 equiv.). The mixture was then stirred at 80°C for 1h, then concentrated in vacuo. The residue was dissolved in THF (15 mL), then sulfuric acid (75 μL) and water (20 μL) were added. After stirring at 20°C for 1h, excess sodium carbonate was added and after stirring for 20 min, the mixture was filtered through celite and concentrated in vacuo. Flash chromatography of the crude led to pure azido-alcool derivatives.

**5.1- (1*S*,2*S*,3*S*,4*S*,6*S*,7*S*)-7-Azido-1,4-di-*O*-*tert*-butyldimethylsilyl-2,3-*O*-methylethylidene-cyclooctane-1,2,3,4,6-pentol (11).**

Isolated yield: 95%; *Rf* 0.2 (cyclohexane/EtOAc 95:5); mp 69-70°C; [α]_D_= +12 (*c*1.0, CH_2_Cl_2_); ^1^H NMR *δ* 4.04 (dd*l*, 1H, *J*_3,2a_= 6.5 Hz, *J*_3,4_= 4.2 Hz, H_3_), 3.94 (dd, 1H, *J*_6,5_= 6.3 Hz, *J*_6,7a_= 1.8 Hz, *J*_6,7b_= 5.5 Hz, H_6_), 3.73 (ddd, 1H, *J*_8,1_= 9.5 Hz, *J*_8,7a_= 8.8 Hz, *J*_8,7b_= 1.5 Hz, H_8_), 3.73 (ddd, 1H, *J*_1,2a_= 2.2 Hz, *J*_1,2b_= 9.0 Hz, *J*_1,8_= 9.5 Hz, H_1_), 3.59 (dd, 1H, *J*_5,4_= 9.5 Hz, *J*_5,6_= 6.3 Hz, H_5_), 3.50 (dd, 1H, *J*_4,3_= 4.2 Hz, *J*_4,5_= 9.5 Hz, H_4_), 2.06 (ddd, 1H, *J*_2a,1_= 2.2 Hz, *J*_2a,2b_= 15.4 Hz, *J*_2a,3_= 6.5 Hz, H_2a_), 2.06 (ddd, 1H, *J*_7a,6_= 1.8 Hz, *J*_7a,7b_= 15.4 Hz, *J*_7a,8_= 8.8 Hz, H_7a_), 1.95 (ddd, 1H, *J*_7b,6_= 5.5 Hz, *J*_7b,7a_= 15.4 Hz, *J*_7b,8_= 1.5 Hz, H_7b_), 1.90 (ddd, 1H, *J*_2b,1_= 9.0 Hz, *J*_2b,2a_= 15.4 Hz, *J*_2b,3_= 1.5 Hz, H_2b_), 1.31 (s, 6H, CH_3_), 0.90, 0.88 (2s, 18H, Si*t*Bu), 0.10, 0.09, 0.08 (3s, 12H, SiMe_2_); ^13^C NMR *δ* 107.3 (CMe_2_), 77.5, 77.3 (C_4,5_), 69.6 (C_8_), 66.0 (C_3,6_), 64.1 (C_1_), 39.4, 36.6 (C_2,7_), 27.2 (CMe_2_), 26.0 (SiC(CH_3_)_3_), 18.2, 18.1 (SiC(CH_3_)_3_), -4.5, -4.8, -4.9, -5.1 (SiMe_2_); HRMS for C_23_H_47_O_5_NSi_2_ (M^+^-N_2_+1): calcd 474.3071; found 474.3065.

**5.2- (1*R*,2*S*,3*S*,4*R*,6*R*,7*R*)-7-Azido-1,4-di-*O*-*tert*-butyldimethylsilyl-2,3-*O*-methylethylidene-cyclooctane-1,2,3,4,6-pentol (12).**

Isolated yield: 98%; *Rf* 0.2 (cyclohexane/EtOAc 95:5); mp 80-83°C; [α]_D_= -33 (*c*1.0, CH_2_Cl_2_); ^1^H NMR *δ* 4.28 (d, 1H, *J*_3,2a_= 8.6 Hz, H_3_), 4.27 (d, 1H, *J*_6,7a_= 8.6 Hz, H_6_), 3.97 (d, 1H, *J*_4,5_= 8.8 Hz, H_4_), 3.94 (d, 1H, *J*_5,4_= 8.8 Hz, H_5_), 3.78 (ddd, 1H, *J*_8,1_= 9.0 Hz, *J*_8,7b_= 8.0 Hz, H_8_), 3.75 (dd, 1H, *J*_1,2b_= 8.0 Hz, *J*_1,8_= 9.0 Hz, H_1_), 2.10 (2dd, 2H, *J*_2a,3_= *J*_7a,6_= 8.6 Hz, *J*_2a,2b_= *J*_7a,7b_= 15.2 Hz, H_2a,7a_), 1.86 (dd, 1H, *J*_2b,2a_= 15.2 Hz, *J*_2b,1_= 8.0 Hz, H_2b_), 1.81 (dd, 1H, *J*_7b,7a_= 15.2 Hz, *J*_7b,8_= 8.0 Hz, H_7b_), 1.34 (s, 6H, CH_3_), 0.92, 0.90 (2s, 18H, Si*t*Bu), 0.11, 0.10, 0.09 (3s, 12H, SiMe_2_); ^13^C NMR *δ* 107.4 (CMe_2_), 77.8, 77.6 (C_4,5_), 69.8 (C_8_), 66.2 (C_3,6_), 64.4 (C_1_), 39.6, 36.9 (C_2,7_), 27.3 (CMe_2_), 26.0, 25.9 (SiC(CH_3_)_3_), 18.2, 18.1 (SiC(CH_3_)_3_), -4.4, -4.8, -5.0, -5.2 (SiMe_2_); HRMS for C_23_H_48_N_3_O_5_Si_2_ (M^+^+1): calcd 502.3133; found 502.3141.

**6- Azido group reduction**

To a suspension of palladium black (100 mg) in ethyl acetate (4 mL) saturated with dihydrogen was added a solution of azido-alcool (1.2 mmol) in ethyl acetate (2 mL). After stirring for 3h, the catalyst was discarded by filtration through celite and the filtrate was concentrated in vacuo. The corresponding aminocyclooctane derivative was isolated as a white solid, and submitted to the reductive amination without purification.

**6.1- (1*S*,2*S*,3*S*,4*S*,6*S*,7*S*)-7-Amino-1,4-di-*O*-*tert*-butyldimethylsilyl-2,3-*O*-methylethylidene-cyclooctane-1,2,3,4,6-pentol (13).**

^1^H NMR *δ* 4.00 (ddd, 1H, *J*_6,5_= 4.7 Hz, *J*_6,7a_= 6.2 Hz, *J*_6,7b_= 1.5 Hz, H_6_), 3.89 (ddd, 1H, *J*_3,2a_= 1.9 Hz, *J*_3,2b_= 5.5 Hz, *J*_3,4_= 5.6 Hz, H_3_), 3.61 (dd, 1H, *J*_4,3_= 5.6 Hz, *J*_4,5_= 9.3 Hz, H_4_), 3.52 (dd, 1H, *J*_5,4_= 9.3 Hz, *J*_5,6_= 4.7 Hz, H_5_), 3.49 (ddd, 1H, *J*_8,1_= 8.5 Hz, *J*_8,7a_= 2.1 Hz, *J*_8,7b_= 7.5 Hz, H_8_), 2.87 (ddd, 1H, *J*_1,2a_= 6.9 Hz,  *J*_1,2b_= 1.6 Hz, *J*_1,8_= 8.5 Hz, H_1_), 2.06 (ddd, 1H, *J*_7a,6_= 6.2 Hz, *J*_7a,7b_= 15.4 Hz, *J*_7a,8_= 2.1 Hz, H_7a_), 1.93 (ddd, 1H, *J*_2a,1_= 6.9 Hz, *J*_2a,2b_= 15.1 Hz, *J*_2a,3_= 1.9 Hz, H_2a_), 1.87 (ddd, 1H, *J*_7b,6_= 1.5 Hz, *J*_7b,7a_= 15.1 Hz, *J*_7b,8_= 7.5 Hz, H_7b_), 1.73 (ddd, 1H, *J*_2b,1_= 1.6 Hz, *J*_2b,2a_= 15.1 Hz, *J*_2b,3_= 5.5 Hz, H_2b_), 1.30 (s, 6H, CH_3_), 0.89, 0.88 (2s, 18H, Si*t*Bu), 0.09, 0.08, 0.06 (3s, 12H, SiMe_2_); ^13^C NMR *δ* 107.6 (CMe_2_), 82.8, 82.0 (C_4,5_), 72.2 (C_8_), 71.5 (C_3_), 70.3 (C_6_), 51.7 (C_1_), 40.9 (C_2_), 38.6 (C_7_), 26.8 (CMe_2_), 25.8 (SiC(CH_3_)_3_), 18.1 (SiC(CH_3_)_3_), -4.7, -5.1 (SiMe_2_); HRMS for C_23_H_50_NO_5_Si_2_ (M^+^+1): calcd 476.3228; found 476.3220.

**6.2- (1*R*,2*S*,3*S*,4*R*,6*R*,7*R*)-7-Amino-1,4-di-*O*-*tert*-butyldimethylsilyl-2,3-*O*-methylethylidene-cyclooctane-1,2,3,4,6-pentol (14).**

^1^H NMR *δ* 4.24 (d*l*, 1H, *J*_6,7b_= 8.9 Hz, H_6_), 4.20 (d*l*, 1H, *J*_3,2a_= 9.5 Hz, H_3_), 3.99 (dd, 1H, *J*_4,3_= 1.3 Hz, *J*_4,5_= 8.7 Hz, H_4_), 3.93 (dd, 1H, *J*_5,4_= 8.7 Hz, *J*_5,6_= 1.3 Hz, H_5_), 3.51 (dd*l*, 1H, *J*_8,1_= 7.8 Hz, *J*_8,7a_= 8.7 Hz, H_8_), 2.93 (dd, 1H, *J*_1,2b_= 8.6 Hz, *J*_1,8_= 7.8 Hz, H_1_), 2.51 (s*l*, 3H, OH_8_, (NH_2_)_1_), 2.16 (ddd, 1H, *J*_7a,6_= 1.1 Hz, *J*_7a,7b_= 14.9 Hz, *J*_7a,8_= 8.7 Hz, H_7a_), 1.94 (dd*l*, 1H, *J*_2a,2b_= 15.2 Hz, *J*_2a,3_= 8.2 Hz, H_2a_), 1.74 (dd, 1H, *J*_2b,1_= 8.6 Hz, *J*_2b,2a_= 15.2 Hz, H_2b_), 1.68 (ddd, 1H, *J*_7b,6_= 8.9 Hz, *J*_7b,7a_= 14.9Hz, *J*_7b,8_= 0.8 Hz, H_7b_), 1.33 (s, 6H, CH_3_), 0.88 (s, 18H, Si*t*Bu), 0.08, 0.07, 0.06, 0.05 (4s, 12H, SiMe_2_); ^13^C NMR *δ* 106.5 (CMe_2_), 78.2 (C_4,5_), 69.6 (C_8_), 67.3 (C_3_), 67.1 (C_6_), 51.1 (C_1_), 43.3 (C_2_), 41.3 (C_7_), 27.3 (CMe_2_), 26.0, 25.9 (SiC(CH_3_)_3_), 18.1 (SiC(CH_3_)_3_), -4.5, -4.9 (SiMe_2_); HRMS for C_23_H_50_NO_5_Si_2_ (M^+^+1): calcd 476.3228; found 476.3223.

**7- Reductive amination**

To the primary amine (0.21 mmol) at 20°C, were successively added titanium(IV) tetra-isopropoxide (0.47 mmol, 2.25 equiv.) and 2,2-dimethyl-1,3-dioxan-5-one (0.32 mmol, 1.5 equiv.). After stirring for 3h, absolute ethanol (0.17 mL) and sodium cyanoborohydride (1.26 mmol, 6 equiv.) were added and the mixture was stirred overnight at 20°C. Addition of water (0.2 mL) resulted in the formation of a white precipitate which was filtered and washed with absolute ethanol. Concentration of the filtrate was followed by extraction of the residue with ethyl acetate, filtration and concentration in vacuo. The oily residue was then dissolved in CH_2_Cl_2_, stirred in the presence of sodium hydrogencarbonate, filtered and concentrated in vacuo. Flash chromatography of the crude led to pure secondary amine.

**7.1- (1*S*,2*S*,3*S*,4*S*,6*S*,7*S*)-7-(2,2-Dimethyl-[1,3]dioxan-5-ylamino)-1,4-di-*O*-*tert*-butyldimethylsilyl-2,3-*O*-methylethylidene-cyclooctane-1,2,3,4,6-pentol (17).**

Isolated yield: 46%; *Rf* 0.3 (cyclohexane/EtOAc 9:1); [α]_D_= +4 (*c*1.0, CH_2_Cl_2_); ^1^H NMR *δ* 3.94 (ddd, 1H, *J*_3,2a_= *J*_3,2b_= 3.1 Hz, *J*_3,4_= 6.9 Hz, H_3_), 3.92 (ddd, 1H, *J*_6,5_= 7.1 Hz, *J*_6,7a_= 3.6 Hz, *J*_6,7b_= 3.4 Hz, H_6_), 3.79 (d, 1H, *J*_1'a,1'b_= 11.7 Hz, H_1'a_), 3.72 (dd, 1H, *J*_4,3_= 6.9 Hz, *J*_4,5_= 7.3 Hz, H_4_), 3.72 (d, 1H, *J*_3'a,3'b_= 11.7 Hz, H_3'a_), 3.67 (dd, 1H, *J*_5,4_= 7.3 Hz, *J*_5,6_= 7.4 Hz, H_5_), 3.59 (d, 1H, *J*_1'b,1'a_= 11.7 Hz, H_1'b_), 3.55 (dd, 1H, *J*_8,1_= 9.2 Hz, *J*_8,7b_= 10.3 Hz, H_8_), 3.54 (d, 1H, *J*_3'b,3'a_= 11.7 Hz, H_3'b_), 3.00 (dd, 1H, *J*_1,2b_= 10.0 Hz, *J*_1,8_= 9.2 Hz, H_1_), 2.12 (dd, 1H, *J*_7a,6_= 3.5 Hz, *J*_7a,7b_= 15.1 Hz, H_7a_), 1.98 (dd, 1H, *J*_2a,2b_= 15.0 Hz, *J*_2a,3_= 3.1 Hz, H_2a_), 1.87 (ddd, 1H, *J*_7b,6_= 3.4 Hz, *J*_7b,7a_= 15.1 Hz, *J*_7b,8_= 10.3 Hz, H_7b_), 1.80 (ddd, 1H, *J*_2b,1_= 10.0 Hz, *J*_2b,2a_= 15.0 Hz, *J*_2b,3_= 3.1 Hz, H_2b_), 1.43, 1.41 (2s, 6H, CH_3_'), 1.32 (s, 6H, CH_3_), 0.88, 0.87 (2s, 18H, Si*t*Bu), 0.09, 0.07, 0.06, 0.05 (4s, 12H, SiMe_2_); ^13^C NMR *δ* 109.4 (CMe_2_), 98.9 (CMe'_2_), 82.2, 82.0 (C_4,5_), 78.8 (C_8_), 74.1 (C_3_), 73.8 (C_6_), 68.0 (C_1'_), 67.4 (C_3'_), 57.3 (C_1_), 37.1 (C_7_), 35.7 (C_2_), 26.9 (CMe_2_), 25.8 (SiC(CH_3_)_3_), 25.0 (CMe'_2_), 18.1 (SiC(CH_3_)_3_), -4.5, -4.9, -5.6 (SiMe_2_); Anal. calcd for C_29_H_59_NO_7_Si_2_: C, 59.04; H, 10.08; N, 2.37; found C, 59.04; H, 10.01; N, 2.38.

**7.2- (1*R*,2*S*,3*S*,4*R*,6*R*,7*R*)-7-(2,2-Dimethyl-[1,3]dioxan-5-ylamino)-1,4-di-*O*-*tert*-butyldimethylsilyl-2,3-*O*-methylethylidene-cyclooctane-1,2,3,4,6-pentol (18).**

Isolated yield: 58%; *Rf* 0.4 (cyclohexane/acetone 85:15); [α]_D_= -33 (*c*1.0, CH_2_Cl_2_); ^1^H NMR *δ* 4.32-4.23 (m, 2H,H_3,6_), 4.04 (d, 1H, *J*_4,5_= 8.4 Hz, H_4_), 3.98 (d, 1H, *J*_5,4_= 8.4 Hz, H_5_), 3.75 (d, 1H, *J*_1'a,1'b_= 11.6 Hz, H_1'a_), 3.71 (dd, 1H, *J*_8,1_= *J*_8,7b_= 9.2 Hz, H_8_), 3.67 (d, 1H, *J*_3'a,3'b_= 11.6 Hz, H_3'a_), 3.56 (d, 1H, *J*_1'b,1'a_= 11.6 Hz, H_1'b_), 3.51 (d, 1H, *J*_3'b,3'a_= 11.6 Hz, H_3'b_), 3.17 (dd, 1H, *J*_1,2b_= *J*_1,8_= 9.2 Hz, H_1_), 2.33 (dd*l*, 1H, *J*_7a,6_= 7.6 Hz, *J*_7a,7b_= 14.5 Hz, H_7a_), 2.21 (dd*l*, 1H, *J*_2a,2b_= 14.1 Hz, *J*_2a,3_= 7.3 Hz, H_2a_), 1.57 (ddd, 1H, *J*_7b,6_= 7.0 Hz, *J*_7b,7a_= 14.5 Hz, *J*_7b,8_= 9.2 Hz, H_7b_), 1.51 (ddd, 1H, *J*_2b,1_= 9.2 Hz, *J*_2b,2a_= 14.1 Hz, *J*_2b,3_= 6.3 Hz, H_2b_), 1.40 (s, 6H, CH_3_'), 1.31 (s, 6H, CH_3_), 0.89, 0.88 (2s, 18H, Si*t*Bu), 0.07 (s, 12H, SiMe_2_); ^13^C NMR *δ* 106.1 (CMe_2_), 98.8 (CMe'_2_), 87.4 (C_2_'), 78.3 (C_8_), 78.2, 78.1 (C_4,5_), 68.1, 67.9 (C_3,6_), 67.7, 67.2 (C_1',3'_), 56.6 (C_1_), 38.9 (C_7_), 37.7 (C_2_), 27.3 (CMe_2_), 26.9 (CMe'_2_), 25.9 (SiC(CH_3_)_3_), 18.1 (SiC(CH_3_)_3_), -4.5, -4.8 (SiMe_2_); HRMS for C_29_H_60_NO_7_Si_2_ (M^+^+1): calcd 590.3908; found 590.3924.

**8- Deprotection of the aminocyclooctanols**

The substrate (0.21 mmol) was stirred in a 9:1 solution of trifluoroacetic acid/H_2_O at 20°C for 15h. After concentration in vacuo, the resulting oily residue was triturated in diethyl ether and the supernatant was discarded to afford a brownish solid which was then purified by ion-exchange chromatography (Dowex® 50x8-100, 1% aqueous ammonium hydroxide).

**8.1-(1*S*,2*R*,3*R*,4*S*,6*S*,7*S*)-7-Amino-cyclooctane-1,2,3,4,6-pentol (15).**

Isolated yield: 95%; [α]_D_ +8 (*c*1.0, H_2_O); ^1^H NMR (D_2_O) *δ* 3.94 (ddd, 1H, *J*_3,2a_= 4.3 Hz, *J*_3,2b_= 7.7 Hz, *J*_3,4_= 8.1 Hz, H_3_), 3.93 (ddd, 1H, *J*_6,5_= *J*_6,7a_= 7.8 Hz, *J*_6,7b_= 3.8 Hz, H_6_), 3.69 (ddd, 1H, *J*_8,1_= 9.6 Hz, *J*_8,7a_= 3.8 Hz, *J*_8,7b_= 6.1 Hz, H_8_), 3.55 (dd, 1H, *J*_4,3_= 8.1 Hz, *J*_4,5_= 8.5 Hz, H_4_), 3.44 (dd, 1H, *J*_5,4_= 8.5 Hz, *J*_5,6_= 7.8 Hz, H_5_), 3.06 (ddd, 1H, *J*_1,2a_= 9.4 Hz, *J*_1,2b_= 3.0 Hz, *J*_1,8_= 9.6 Hz, H_1_), 2.20-2.05 (m, 3H, H_2a,7a,7b_), 1.92 (ddd, 1H, *J*_2b,1_= 3.1 Hz, *J*_2b,2a_= 15.3 Hz, *J*_2b,3_= 7.7 Hz, H_2b_); ^13^C NMR (D_2_O) *δ* 77.8, 77.4 (C_4,5_), 72.9 (C_8_), 71.2, 70.9 (C_3,6_), 55.4 (C_1_), 40.7 (C_7_), 40.1 (C_2_); HRMS for C_8_H_18_O_5_N (M^+^+1): calcd 208.1185; found 208.1186.

**8.2- (1*R*,2*R*,3*R*,4*R*,6*R*,7*R*)-7-Amino-cyclooctane-1,2,3,4,6-pentol (16).**

Isolated yield: 68%; [α]_D_ -15 (*c*1.0, H_2_O); ^1^H NMR (D_2_O) *δ* 4.24 (2ddd, 2H, *J*_3,2a_= *J*_6,7b_= 7.1 Hz, *J*_3,2b_= *J*_6,7a_= 2.5 Hz, *J*_3,4_= *J*_6,5_= 1.4 Hz, H_3,6_), 3.96 (dd, 1H, *J*_5,4_= 7.1 Hz, *J*_5,6_= 1.4 Hz, H_5_), 3.91 (dd, 1H, *J*_4,3_= 1.4 Hz, *J*_4,5_= 7.1 Hz, H_4_), 3.78 (ddd, 1H, *J*_8,1_= 9.1 Hz, *J*_8,7a_= 6.2 Hz, *J*_8,7b_= 3.4 Hz, H_8_), 3.16 (ddd, 1H, *J*_1,2a_= 3.4 Hz, *J*_1,2b_= 7.1 Hz, *J*_1,8_= 9.1 Hz, H_1_), 2.10 (ddd, 1H, *J*_7a,6_= 2.5 Hz, *J*_7a,7b_= 15.5 Hz, *J*_7a,8_= 6.2 Hz, H_2b_), 2.08-1.80 (m, 3H, H_2a,2b,7b_); ^13^C NMR (D_2_O) *δ* 75.2 (C_8_), 71.8 (C_4,5_), 68.6 (C_3,6_), 54.3 (C_1_), 38.7 (C_7_), 35.5 (C_2_); HRMS for C_8_H_18_O_5_N (M^+^+1): calcd 208.1185; found 208.1183.

**8.3- (1*S*,2*R*,3*R*,4*S*,6*S*,7*S*)-7-(2-Hydroxy-1-hydroxymethyl-ethylamino)-cyclooctane-1,2,3,4,6-pentol (19).**

Isolated yield: 100%; [α]_D_ +14 (*c*1.0, H_2_O); ^1^H NMR (D_2_O) *δ* 3.88-3.70 (m, 2H, H_3,6_), 3.95-3.40 (m, 4H, H_1',3'_), 3.55 (ddd, 1H, *J*_8,1_= 9.8 Hz, *J*_8,7a_= 5.5 Hz, *J*_8,7b_= 4.8 Hz, H_8_), 3.40 (dd, 1H, *J*_4,3_= *J*_4,5_= 8.3 Hz, H_4_), 3.31 (dd, 1H, *J*_5,4_= *J*_5,6_= 8.3 Hz, H_5_), 2.87 (ddd, *J*_1,2a_= 4.1 Hz, *J*_1,2b_= 3.3 Hz, *J*_1,8_= 9.8 Hz, H_1_), 2.20-2.00 (m, 2H, H_7a,7b_), 1.94 (ddd, 1H, *J*_2a,1_= 4.1 Hz, *J*_2a,2b_= 15.3 Hz, *J*_2a,3_= 8.2 Hz, H_2a_), 1.80 (ddd, *J*_2b,1_= 3.3 Hz, *J*_2b,2a_= 15.3 Hz, *J*_2b,3_= 7.8 Hz, H_2b_); ^13^C NMR (D_2_O) *δ* 79.7, 77.8 (C_4,5_), 75.0 (C_8_), 71.4 (C_3,6,1',3'_), 53.6 (C_1_), 42.0 (C_7_), 40.8 (C_2_); HRMS for C_11_H_24_NO_7_ (M^+^+1): calcd 282.1553; found 282.2779.

**8.4- (1*R*,2*R*,3*R*,4*R*,6*R*,7*R*)-7-(2-Hydroxy-1-hydroxymethyl-ethylamino)-cyclooctane-1,2,3,4,6-pentol (20).**

Isolated yield: 50%; [α]_D_ -1 (*c*1.0, H_2_O); ^1^H NMR (D_2_O) *δ* 4.22-4.08 (m, 2H, H_3,6_), 3.95-3.40 (m, 7H, H_4,5,8,1',3'_), 3.20-3.10 (m, 1H, H_1_), 2.10-1.70 (m, 4H, H_2a,2b,7a,7b_); ^13^C NMR (D_2_O) *δ* 75.4 (C_8_), 74.8, 73.8 (C_4,5_), 69.6 (C_3,6_), 54.3 (C_1_), 39.4 (C_7_), 37.5 (C_2_); HRMS for C_11_H_24_NO_7_ (M^+^+1): calcd 282.1553; found 282.1556.

**9- Inhibition studies**

The inhibition studies were performed according to the procedure previously described. ^40,41^ In the specific case of α-D-glucosidase from *Bacillus stearothermophilus*, β-D-glucosidase from almonds, α-D-mannosidase from Jack beans and α-L-fucosidase from bovine kidney, the biological tests have been carried out as explained in reference 19. Whatever the conditions were, we verified that the delay of inhibtor/enzyme incubation did not affect the inhibition measurements. Under standard conditions optimal inhibitory activities were measured after five minutes of incubation.

**10- X-ray crystallography**

Diffraction data for compounds **7** and **9** were recorded at the LURE synchrotron facility in Orsay, at 100(1) K. The wavelength was fixed to 0.964 Å the detector was a MAR RESEARCH 345 image plate. Both structures were solved using SHELXS and anisotropically refined using SHELXL programs.^38^ **7**: Space group P2_1_2_1_2_1_; a=6.729(1); b=15.246(2); c=27.044(2) Å and Z=4. Final *R* = 0.041 for 1490 observed *Fs* and *R* = 0.043 for all data (2010 *Fs*). **9**: Space group P2_1_; a=12.259(1); b=10.990(1); c=22.990(2) Å; β=90.86(4)° and Z=4. Final *R* = 0.050 for 2937 observed *Fs* and *R* = 0.052 for all data (3030 *Fs*). Structure data were deposited with the Cambridge Crystallography Data Centre under ref. CCDC 266247 and CCDC 266248 respectively.
